# Association between oral health and frailty: results from the Korea National Health and Nutrition Examination Survey

**DOI:** 10.1186/s12877-022-02968-x

**Published:** 2022-04-27

**Authors:** Hyunjoo Kim, Euni Lee, Seok-Woo Lee

**Affiliations:** 1 College of Pharmacy & Research Institute of Pharmaceutical Sciences, Seoul National University, 1 Gwanak-ro, Gwanak-gu, Seoul, 08826 Republic of Korea; 2 Department of Dental Education and Periodontology, School of Dentistry, Dental Science Research Institute, Chonnam National University, Gwangju, 61186 Republic of Korea

**Keywords:** Frailty, Oral health, Health services for the aged, Tooth loss, Periodontal diseases

## Abstract

**Background:**

Previous research has suggested that poor oral health is positively associated with frailty. The objective of this study was to explore associations of key oral diseases (periodontal disease, tooth loss), and oral hygiene and management behaviors with the level of frailty in community-dwelling older Korean adults using national representative survey data.

**Methods:**

This study used cross-sectional, 6th and 7th Korea National Health and Nutrition Examination Survey (KNHANES VI, VII) data. Adults aged 50+ years were included. Frailty was measured using frailty phenotype (FP) and frailty index (FI). FP was determined using five frailty criteria, i.e., weight loss, weakness, exhaustion, slowness, or low physical activity, and the level of frailty was classified with the number of criteria present (*robust*, none; *pre-frail,* 1–2; *frail*, 3+). FI was determined using a 44-item FI constructed according to a standard protocol, and the level of frailty was classified as *robust* (FI: ≤ 0.08), *pre-frail* (FI: 0.08–0.25), and *frail* (FI: ≥ 0.25). Multiple ordinal regression analyses were conducted with each type of frailty as the outcome variable. Independent variables of interest were the periodontal status, number of teeth, and practices on oral hygiene and management. Analyses were additionally adjusted for participants’ socioeconomic, diet, and behavioral characteristics.

**Results:**

The prevalence of frailty was 4.38% according to the FP classification (*n* = 4156), 10.74% according to the FI classification (*n* = 15,073). In the final adjusted model, having more teeth and brushing after all three meals were significantly associated with lower odds of being more frail (in both frailty models); no significant association was observed between periodontal disease and frailty.

**Conclusions:**

Findings from this study show having more teeth and practicing adequate brushing are significantly associated with frailty. Due to limitations of the study design, well-designed longitudinal studies are needed to confirm these findings.

**Supplementary Information:**

The online version contains supplementary material available at 10.1186/s12877-022-02968-x.

## Background

Frailty is defined as a state of increased vulnerability to stressors due to decreased functional reserves and impaired homeostasis across multiple physiological systems [1–4]. It is a major concern in this aging society because it is associated with higher risks for adverse health outcomes including falls, hospitalization, and death [1–4]. Well-known risk factors for frailty include old age, female sex, lower socioeconomic status, and a number of comorbid chronic illnesses [2]. Among well-acknowledged definitions of frailty, the definition of Fried’s frailty phenotype, i.e., physical frailty, and Rockwood frailty index (FI) have been frequently used in previous research [5]. The phenotypic definition views frailty as combined manifestations of a specified set of five criteria, i.e., weight loss, weakness, exhaustion, slow gait, or low physical activity [2]. The FI approach views frailty as an accumulation of an unspecified set of health deficits that can be any disease, disabilities, signs, symptoms, and laboratory abnormalities present in an individual [6].

The reported prevalence of frailty varies across different populations and settings. It also varies depending on the definition of frailty adopted. In a study using the United States (US) National Health and Nutrition Examination Survey (NHANES) data, the prevalence of frail phenotype in adults aged 50 years or more was 3.6% [5]. In the same study, the prevalence of frailty measured using the FI was 34% [5]. The underlying etiology of frailty remains unclear. However, illnesses and conditions associated with frailty frequently involve malnutrition, musculoskeletal defects, and physiological pathways related to insulin resistance and inflammation [7].

Increasing evidence suggests that there is a positive relationship between poor oral health and frailty [8–10]. Several pathways have been postulated to explain this association which includes physiological (e.g., chronic inflammation), functional (e.g., malnutrition), and psychosocial (e.g., negative self-esteem, quality of life) pathways [11]. One of the most common forms of oral diseases is periodontal disease (PD), the progressive destruction of periodontal tissues due to chronic, bacterial inflammation [12]. PD has been linked to various systemic diseases including cardiovascular, neurodegenerative diseases, and cancer, possibly via inflammatory pathways [12, 13]. Severe forms of PD can also lead to tooth loss [14, 15], which has been linked to malnutrition and low physical function [8, 16, 17], well-known risk factors for frailty.

Despite the growing interest in the association between poor oral health and frailty, very few studies have reported the association in South Korea. Such studies using national-level population data are even fewer. Therefore, the objective of this study was to investigate the association between poor oral health and frailty, using both definitions of frailty. We focused our investigation on the two key oral diseases, i.e., PD and tooth loss, and oral hygiene and management practices while adjusting for participants’ socioeconomic, diet, and behavioral characteristics, to gain a comprehensive understanding of the association.

## Methods

### Data source and study population

**Fig. 1:**
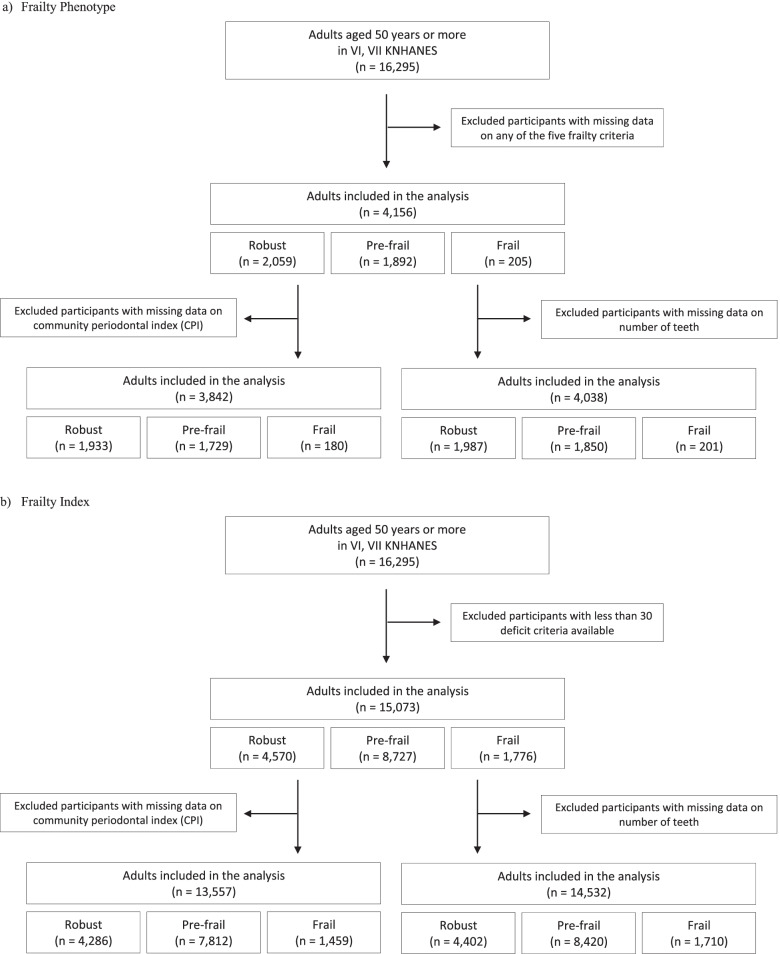
Flowchart showing participant selection. **a** Frailty Phenotype, **b** Frailty Index

This study used the 6th (2013–2015) and 7th (2016–2018) Korea National Health and Nutrition Examination Survey (KNHANES) data. KNHANES is a cross-sectional, national representative survey conducted by the Korea Disease Control and Prevention Agency (KDCA) to examine the general health and nutritional status of non-institutionalized Korean citizens [18]. Survey data used a multi-stage clustered probability sampling design, which allowed sample weights to be extrapolated to national level estimates [18]. The study population included survey participants aged 50 years or more, who had complete frailty data and received oral examination (Fig. 1).

Written informed consents to participate in KNHANES were obtained from the participants and the survey collection process was approved by the KDCA Research Ethics Review Committee. The committee operates based on relevant domestic and international regulations and guidelines, including the Declaration of Helsinki and the Bioethics and Safety Act. Personal data from the survey are de-identified before they are made publicly available. The protocol of our study was approved by the Institutional Review Board (IRB) of Chonnam National University Dental Hospital (approval number: CNUDH-EXP-2021-004).

### Outcome variable

The outcome variable of this study was frailty, as evaluated by frailty phenotype (FP) and frailty index (FI). FP, classified as *robust*, *pre-frail*, and *frail*, was operationally defined as having reported weight loss, weakness, exhaustion, slowness, or low physical activity [2]. The level of frailty was determined using the number of criteria present (*robust*, none; *pre-frail,* 1–2; *frail*, 3 or more criteria) described in Supplementary Table 1 using data elements available in KNHANES data similar to other published studies [5, 19]. FP was considered missing for participants who had missing values for any of the five criteria; survey data from the years 2014, 2016, and 2018 were used because information on all five criteria was complete only in these years.

Frailty was also operationally defined using the FI, a ratio of health deficits present to the total number of health deficits considered, i.e., 44 deficit criteria described in Supplementary Table 2 selected and scored on a scale of 0 (absence of a deficit) to 1 (full expression of the deficit), according to a standard protocol [6] using data elements available in KNHANES data similar to other published studies [5, 19–21]. The level of frailty was determined using previously reported cut-off values (*robust,* FI ≤ 0.08; *pre-frail*, 0.08–0.25; *frail,* FI ≥ 0.25) [21]. Deficits with a prevalence of less than 1% (e.g., tuberculosis, hepatitis C, cirrhosis) and those with a missing value for more than 20% of the study population (e.g., history of hospitalization) were not considered [6, 11, 20]. Variables from the oral examination were not included to avoid overestimating the association between oral health and frailty. FI was considered missing for participants who had less than 30 deficit criteria available [6].

### Independent variables 

PD was defined if World Health Organization (WHO) community periodontal index (CPI) was equal to or higher than 3 (periodontal pocket depth, 4–5 mm) [22]. The periodontal examination was conducted in sextants (tooth numbers 18–14, 13–23, 24–28, 48–44, 43–33, and 34–38), three each in maxillary and mandibular arches; in total, ten index teeth (tooth numbers 17, 16, 11, 26, 27, 47, 46, 31, 36, and 37) were examined if there were two or more natural teeth present in the sextant [22, 23]. The maximum CPI value among all sextants was recorded [22, 23]. The examination was performed using CPI probes fulfilling WHO requirements, and a probing force of ≈20 g was applied [22]. The total number of teeth was calculated by summing up all non-missing, natural teeth excluding wisdom teeth (range: 0 to 28) [24]. Oral hygiene and management behaviors were additionally collected via interviews. Participants were asked about their brushing patterns from the previous day, such as the time of brushing relative to meals and the tools used, and the history of oral examinations and treatments within the previous year. All oral examinations were performed by licensed dentists who’ve completed KNHANES simulation training programs [22, 24].

### Statistical analysis

The study analysis focused on estimating the prevalence of frailty and evaluating its associated factors. To obtain national-level estimates (i.e., weighted estimates), assigned sampling weights were applied to reflect the complex sampling design [18]. Descriptive statistics and multiple ordinal regression analyses were performed using weighted data. Ordinal regression analysis provides pooled results by grouping *pre-frail* and *frail* groups (versus the *robust* group) and *pre-frail* and *robust* groups (versus *frail* group) [25]. The final odds ratio (OR) allows an inference between lower and higher levels of the outcome variable, frailty [25]. Variables with *p*-values < 0.05 from the univariable analysis were included in regression analyses, and adjusted OR and 95% confidence interval (CI) were determined. All statistical analyses were conducted using SAS version 9.4 (SAS Institute Inc., Cary, NC, USA). A two-sided alpha level of *p* <  0.05 was used to determine statistical significance.

## Results

The prevalence of frailty was 4.38% according to the FP classification (*n* = 4156) and 10.74% according to the FI classification (*n* = 15,073) (Fig. 1, Table 1). In the unadjusted analysis for general characteristics and frailty (Table 1), compared to the *robust* group, participants in *pre-frail* or *frail* groups were significantly older, and a significantly higher percentage of participants were female, on a poor diet, of lower socioeconomic status, and had more comorbidities in both frailty models; regarding the behavioral characteristics, compared to the *robust* group, a significantly lower percentage of participants in *pre-frail* or *frail* groups had a history of smoking or were frequent drinkers in both frailty models (except for smoking history which was not significant in the FP model, *p* = 0.118).

**Table 1:** General characteristics stratified by frailty level (age ≥ 50 years)

Characteristics		Frailty Phenotype			***p***	Frailty Index			***p***
		Robust	Pre-frail	Frail		Robust	Pre-frail	Frail	
Total participants		2059 (53.79)	1892 (41.83)	205 (4.38)		4570 (33.23)	8727 (56.02)	1776 (10.74)	
Age (years), weighted mean (SE)		59.37 (0.25)	63.90 (0.32)	68.93 (0.89)	p1: < 0.001 p2: < 0.001	58.31 (0.13)	64.07 (0.14)	71.39 (0.28)	p1: < 0.001 p2: < 0.001
Sex	Males	939 (49.57)	829 (45.12)	78 (32.43)	< 0.001	2124 (49.34)	3727 (44.35)	554 (32.05)	< 0.001
	Females	1120 (50.43)	1063 (54.88)	127 (67.57)		2446 (50.66)	5000 (55.65)	1222 (67.95)	
Diet quality	Not poor	743 (56.07)	598 (50.36)	64 (48.01)	0.014	2138 (58.24)	3906 (55.04)	807 (54.38)	0.007
	Poor^a^	1316 (43.93)	1294 (49.64)	141 (51.99)		2432 (41.76)	4821 (44.96)	969 (45.62)	
Living in rural areas	No	1093 (53.35)	915 (47.57)	97 (48.42)	0.033	2209 (48.60)	3972 (45.02)	755 (41.85)	0.001
	Yes^b^	966 (46.65)	977 (52.43)	108 (51.58)		2361 (51.40)	4755 (54.98)	1021 (58.15)	
Low household income	No	1216 (62.01)	771 (44.25)	41 (22.99)	< 0.001	2797 (63.36)	3403 (42.41)	324 (20.13)	< 0.001
	Yes^c^	837 (37.99)	1116 (55.75)	163 (77.01)		1760 (36.64)	5280 (57.59)	1446 (79.87)	
Education (years)	≥ 9 years	1629 (80.13)	1092 (60.96)	86 (47.99)	< 0.001	3581 (80.22)	4749 (56.91)	472 (28.88)	< 0.001
	< 9 years	430 (19.87)	796 (39.04)	119 (52.01)		971 (19.78)	3914 (43.09)	1268 (71.12)	
Living alone	No	1728 (85.15)	1419 (77.79)	125 (61.73)	< 0.001	3910 (86.38)	6446 (75.39)	926 (51.84)	< 0.001
	Yes^d^	330 (14.85)	472 (22.21)	80 (38.27)		656 (13.62)	2275 (24.61)	849 (48.16)	
Type of national health insurance	Self/workplace	2030 (98.41)	1789 (94.81)	178 (85.86)	< 0.001	4496 (98.42)	8200 (94.08)	1423 (80.94)	< 0.001
	Others^e^	29 (1.59)	103 (5.19)	27 (14.14)		74 (1.58)	527 (5.92)	353 (19.06)	
Smoking history	Never	1308 (60.81)	1166 (61.10)	139 (70.59)	0.118	2809 (59.99)	5331 (59.97)	1152 (66.38)	< 0.001
	Past/current^f^	750 (39.19)	723 (38.90)	65 (29.41)		1736 (40.01)	3289 (40.03)	558 (33.62)	
Frequent alcohol drinking	No	648 (30.58)	743 (37.03)	103 (52.76)	< 0.001	1331 (28.41)	3502 (38.36)	1077 (62.10)	< 0.001
	Yes^g^	1411 (69.42)	1147 (62.97)	102 (47.24)		3217 (71.59)	5129 (61.64)	641 (37.90)	
Number of comorbidities^h^	< 2	1305 (65.96)	942 (54.30)	68 (31.69)	< 0.001	3966 (87.57)	3518 (41.83)	408 (26.17)	< 0.001
	≥ 2	754 (34.04)	950 (45.70)	137 (68.31)		604 (12.43)	5209 (57.17)	1368 (73.83)	

Unweighted frequencies (weighted percentage) shown, otherwise indicated. Missing data not shown. *p*-values from chi-square test or ANOVA (*p*1: robust vs. pre-frail; *p*2: robust vs. frail). SE: Standard error

^a^ Poor if Korean healthy eating index < 50 (out of 100), a higher score indicating a healthier diet

^b^ Non-metropolitan statistical areas (MSA). MSA were namely Busan, Daegu, Incheon, Gwangju, Daejeon, Sejong, Seoul, and Ulsan

^c^ Equivalised household income i.e., household income/√(number of household members), in the lowest 50%. Quartiles were stratified by sex and age group

^d^ Never married, separated, widowed, or divorced

^e^ Medical aid class 1 or 2, no health insurance, or unknown

^f^ Smoked at least 5 packs of cigarettes in a lifetime

^g^ Drank at least once a month within the past year

^h^ Hypertension, diabetes, dyslipidemia (namely abnormal blood cholesterol, high-density lipoprotein, triglycerides, or low-density lipoprotein), stroke, heart disease (namely myocardial infarction or angina pectoris), arthritis, depression, asthma, cirrhosis, renal failure, and cancer other than skin cancer

In the unadjusted analysis for PD and frailty (Table 2), compared to the *robust* group, a significantly higher percentage of participants in *pre-frail* or *frail* groups had PD in the FI model (*p* = 0.023); no significant association between PD and frailty was observed in the FP model (*p* = 0.234).

**Table 2:** Oral health-related characteristics stratified by frailty level (age ≥ 50 years)

Characteristics		Frailty Phenotype			***p***	Frailty Index			***p***
		Robust	Pre-frail	Frail		Robust	Pre-frail	Frail	
Total participants		2059 (53.79)	1892 (41.83)	205 (4.38)		4570 (33.23)	8727 (56.02)	1776 (10.74)	
***Key oral diseases***									
Periodontal disease	No	1116 (54.98)	987 (58.43)	93 (55.46)	0.234	2477 (56.72)	4194 (53.66)	808 (53.24)	0.023
	Yes	817 (45.02)	742 (41.58)	87 (44.54)		1809 (43.28)	3618 (46.34)	651 (46.76)	
Natural teeth, weighted mean (SE)		23.82 (0.18)	20.99 (0.26)	18.26 (0.78)	p1: < 0.001 p2: < 0.001	23.77 (0.12)	20.72 (0.12)	16.78 (0.28)	p1: < 0.001 p2: < 0.001
***Oral hygiene/management behaviors***									
Brushed after breakfast, lunch, dinner^a^	No	1341 (65.56)	1383 (74.95)	147 (71.54)	< 0.001	3117 (68.54)	6613 (76.03)	1415 (79.38)	< 0.001
	Yes	718 (34.44)	509 (25.05)	58 (28.46)		1453 (31.46)	2114 (23.97)	361 (20.62)	
Brushed before bed^a^	No	1314 (65.56)	1279 (66.96)	152 (72.75)	0.279	2928 (63.67)	6012 (68.20)	1339 (74.34)	< 0.001
	Yes	745 (34.44)	613 (33.04)	53 (27.25)		1641 (36.33)	2714 (31.80)	437 (25.66)	
Currently using floss	No	1659 (82.86)	1629 (85.56)	192 (93.26)	0.006	3696 (82.21)	7552 (87.41)	1614 (94.14)	< 0.001
	Yes	399 (17.14)	260 (14.44)	12 (6.74)		846 (17.79)	1062 (12.59)	96 (5.86)	
Currently using interdental toothbrush	No	1761 (84.47)	1677 (88.94)	179 (88.61)	0.003	3780 (82.06)	7458 (85.76)	1530 (89.96)	< 0.001
	Yes	297 (15.53)	212 (11.06)	25 (11.39)		762 (17.94)	1156 (14.24)	180 (10.04)	
Currently using mouthwash	No	1375 (67.78)	1457 (78.60)	180 (85.47)	< 0.001	3292 (73.26)	6863 (79.75)	1527 (88.77)	< 0.001
	Yes	683 (32.22)	432 (21.40)	24 (14.53)		1250 (26.74)	1751 (20.25)	183 (11.23)	
Currently using electronic toothbrush	No	1958 (94.58)	1823 (96.93)	201 (99.05)	0.001	4318 (94.78)	8330 (96.45)	1669 (97.67)	< 0.001
	Yes	100 (5.42)	66 (3.07)	3 (0.95)		224 (5.22)	284 (3.55)	41 (2.33)	
Currently using other oral hygiene tools^b^	No	1921 (93.45)	1753 (93.41)	193 (94.63)	0.869	4300 (94.88)	8002 (93.33)	1563 (91.94)	0.001
	Yes	137 (6.55)	136 (6.59)	11 (5.37)		242 (5.12)	612 (6.67)	147 (8.06)	
Any oral examination within 1 year^c^	At least once	829 (37.66)	572 (29.66)	43 (21.15)	< 0.001	1710 (36.78)	2427 (28.46)	286 (17.29)	< 0.001
	None	1228 (62.34)	1317 (70.34)	161 (78.85)		2831 (63.22)	6183 (71.54)	1422 (82.71)	
Any prophylactic treatment within 1 year^d^	At least once	688 (29.39)	503 (23.68)	41 (22.28)	0.003	1345 (27.70)	2074 (23.52)	261 (14.09)	< 0.001
	None	1369 (70.61)	1385 (76.32)	163 (77.72)		3196 (72.30)	6537 (76.48)	1443 (85.91)	

Unweighted frequencies (weighted percentage) shown, otherwise indicated. Missing data not shown. *p*-values from chi-square test or ANOVA (*p*1: Robust vs. Pre-frail; *p*2: Robust vs. Frail)

SE: Standard error

^a^ Brushing patterns from the previous day. If the participant did not take a meal but brushed, it was recorded as brushed before meal

^b^ Water pick, tongue cleaner, specialized brush (e.g., toothbrush for implants), denture cleansers, etc.

^c^ Received any regular oral examinations within the past year, even if the participant was not experiencing any problems

^d^ Dental sealants, fluoride treatment, scaling, etc.

In the unadjusted analysis for the number of natural teeth and frailty (Table 2), compared to the *robust* group, participants in *pre-frail* or *frail* groups had significantly fewer teeth in both frailty models.

In the unadjusted analysis for oral hygiene/management behaviors and frailty (Table 2), compared to the *robust* group, a significantly lower percentage of participants in *pre-frail* or *frail* groups were practicing oral hygiene/management in both frailty models (except for the use of other oral hygiene tools which was not significant in the FP model, *p* = 0.869; the percentage of users was significantly higher in *pre-frail* or *frail* groups in the FI model, *p* = 0.001).

In the adjusted regression analysis, no significant association was observed between PD and frailty, in both frailty models (Model 1, Table 3).

**Table 3:** Adjusted ordinal regression model for periodontal disease and frailty (age ≥ 50 years)

Characteristics	Frailty Phenotype	Frailty Index
	Model 1	Model 1
Periodontal disease	0.899 (0.743–1.088)	1.090 (0.990–1.200)
Older	**1.074 (1.061–1.087)**	**1.108 (1.102–1.114)**
Female	**1.433 (1.185–1.733)**	**1.414 (1.290–1.550)**
Poor diet quality	**1.250 (1.037–1.527)**	1.059 (0.969–1.157)

Adjusted odds ratio (95% confidence interval) for frailty (outcome variable) shown. Variables with *p* < 0.05 from the descriptive analysis were included (excluded if frequency < 10 in any cell). Bold values denote statistical significance

In the final adjusted regression analysis (Model 3, Table 4), for every additional tooth, the odds of being more frail was significantly lower in both frailty models, by 0.981 (95% CI, 0.969–0.992; FP) and 0.989 (95% CI, 0.983–0.996; FI). Brushing after all three meals was significantly associated with lower odds of being more frail in both frailty models, by 0.790 (95% CI, 0.651–0.958; FP) and 0.842 (95% CI, 0.755–0.938; FI). Additionally, older age, female sex, fewer years of education, having no self- or workplace- insurance, and having more comorbidities were all significantly associated with higher odds of being more frail, in both frailty models. Diet quality and the use of mouthwash were significant only in the FP model (Table 4).

**Table 4:** Adjusted ordinal regression models for number of natural teeth and frailty (age ≥ 50 years)

Characteristics	Frailty Phenotype			Frailty Index		
	Model 1	Model 2	Model 3	Model 1	Model 2	Model 3
More number of natural teeth	**0.971 (0.960–0.981)**	**0.978 (0.967–0.989)**	**0.981 (0.969–0.992)**	**0.980 (0.974–0.986)**	**0.988 (0.981–0.994)**	**0.989 (0.983–0.996)**
Older	**1.066 (1.053–1.079)**	**1.049 (1.035–1.063)**	**1.048 (1.035–1.062)**	**1.101 (1.095–1.108)**	**1.066 (1.059–1.074)**	**1.066 (1.059–1.073)**
Female	**1.468 (1.224–1.760)**	**1.325 (1.088–1.614)**	**1.349 (1.106–1.646)**	**1.413 (1.293–1.543)**	**1.421 (1.228–1.645)**	**1.448 (1.250–1.678)**
Poor diet quality	**1.255 (1.041–1.513)**	**1.253 (1.042–1.507)**	**1.307 (1.089–1.568)**	1.054 (0.967–1.149)	0.989 (0.908–1.078)	0.999 (0.916–1.090)
Living in rural areas		**1.332 (1.081–1.641)**	**1.312 (1.068–1.611)**		1.076 (0.997–1.184)	1.063 (0.966–1.170)
Low household income		**1.247 (1.013–1.534)**	1.198 (0.970–1.478)		**1.215 (1.086–1.360)**	**1.189 (1.061–1.331)**
Education, < 9 years		**1.538 (1.246–1.900)**	**1.435 (1.159–1.777)**		**1.585 (1.417–1.773)**	**1.510 (1.347–1.692)**
Living alone		0.998 (0.792–1.256)	0.977 (0.775–1.233)		**1.285 (1.134–1.456)**	**1.273 (1.123–1.443)**
No self/workplace insurance		**3.467 (2.134–5.632)**	**3.452 (2.137–5.577)**		**3.293 (2.616–4.144)**	**3.233 (2.565–4.074)**
Past/current smoker					**1.345 (1.161–1.557)**	**1.341 (1.156–1.555)**
Frequent alcohol drinking		0.970 (0.802–1.172)	0.961 (0.792–1.165)		**0.778 (0.704–0.860)**	**0.778 (0.704–0.860)**
Comorbidities, ≥ 2		**1.363 (1.131–1.644)**	**1.357 (1.127–1.635)**		**6.710 (5.994–7.510)**	**6.699 (5.984–7.499)**
Brushed after all three meals			**0.790 (0.651–0.958)**			**0.842 (0.755–0.938)**
Brushed before sleep						0.912 (0.824–1.010)
Floss			1.042 (0.804–1.352)			0.978 (0.852–1.123)
Interdental toothbrush			0.836 (0.637–1.097)			0.999 (0.880–1.133)
Mouthwash			**0.793 (0.633–0.993)**			0.930 (0.826–1.047)
Electronic brush						0.873 (0.698–1.091)
Other oral hygiene tools						1.005 (0.838–1.206)
No oral examination			1.115 (0.911–1.366)			1.056 (0.945–1.181)
No prophylactic dental treatment			1.120 (0.904–1.387)			1.050 (0.936–1.179)

Adjusted odds ratio (95% confidence interval) for frailty (outcome variable) shown. Variables with *p* < 0.05 from the descriptive analysis were included (excluded if frequency < 10 in any cell). Bold values denote statistical significance

## Discussion

Main findings from our study using national-level population big data showed that having more teeth and practicing adequate oral hygiene were negatively associated with frailty in community-dwelling older adults aged 50 years or more in Korea.

The strength of our study was that we were able to provide national-level estimates on the prevalence of frailty and its associated factors. Numerous studies have previously assessed the link between oral health and frailty; however, they were mostly conducted using site- or community-level data [8]. Second, all oral examinations were conducted by licensed dentists. Lastly, the analysis population included a slightly younger population compared to the previous studies where adults were usually at least 65 years old [5, 17]. This study also has some limitations, primarily due to the cross-sectional design of the survey limiting the causality assessment. Second, the nature of KNHANES as a secondary dataset. Lastly, the use of a number of self-reported variables might have a recall bias.

In this study, two well-acknowledged definitions of frailty were used. To the best of our knowledge, this is the first study to assess the association between oral health and frailty using both definitions of frailty in a single study. This allows interpretation of the results in contexts of different types of frailty simultaneously. It is well known that the two definitions of frailty are different; therefore, should be considered complementary [26]. Furthermore, this study was one of the few studies using secondary data that used all five criteria for assessing frailty phenotype, as most of the similar studies have so far used a 4-item version of the definition [5].

Due to our analytic approach where participants had to have all five frailty criteria to estimate FP, more than 70% of the eligible age group was excluded from the FP analysis. When the general characteristics of the included and excluded participants were compared (Supplementary Table 3), we found that excluded participants were older, more frail, and in general, of lower socioeconomic status. This may have compromised the generalizability of the study results; however, such limitation was partially compensated by conducting the FI analysis in parallel in which only ≈9% of the eligible age group was excluded.

In this study, we could not find a significant association between PD and frailty. Previous studies that have evaluated the association have also shown inconsistent results [11]. The lack of association may root in using CPIs to classify periodontal status. Previous studies have reported that CPIs may not provide a whole picture of an individual’s periodontal status due to the use of index teeth as in our study where only the ten index teeth were examined at the most [27–29]. Moreover, because CPIs do not measure clinical attachment loss, they may underestimate the prevalence of PD [27–29]. Also, the high prevalence of PD across all levels of frailty in our study (more than 40%, in both frailty models) may have affected the results from our analyses.

In all adjusted models, each additional tooth and adequate brushing were significantly associated with lower odds of being more frail, even after adjusting for diet quality. Of note, poor diet quality was positively associated with frailty only in the FP model, which is in line with previous studies where weaker associations were observed between nutritional factors and FI compared to FP [10, 11]. Findings from this study were confirmed through a series of sensitivity analyses: by modifying the operational definition of weight loss criterion in FP assessment to that frequently used in studies using NHANES data [5], and by changing the analysis population to adults at least 65 years old (Supplementary Tables 4 and 5).

The national-level estimate on the prevalence of frailty was 4.38% using the phenotypic approach and 10.74% using the FI, values well within the range of those previously reported [5, 30, 31]. As the chance of recovery from frailty declines with age, it is important to identify individuals who are at higher risk of becoming frail to provide early interventions which can help sustain independence and maximize functional capabilities for as long as possible [32–34]. As the proportion of the aged population continues to rise, various forms of interdisciplinary collaboration among community healthcare providers are increasing [35]. From these models, we could consider expanding the roles of community pharmacists who are well-positioned both physically and psychologically for communicating with community-dwelling older adults [35]. Their roles can be expanded to counseling about various products for oral hygiene, detecting and referring older adults with poor oral health, and supporting the management of community oral health and hygiene in collaboration with local dental clinics. In addition, efforts to increase public awareness on the importance of oral health and maintaining robustness can be considered.

Due to limitations of the study design, well-designed longitudinal studies are needed to confirm the study findings. Also, future studies should elaborate on the association between oral health and frailty which can include psychosocial elements in the older population. Despite limitations, results from our study provide a comprehensive understanding of the associations between oral health and frailty which are valuable in setting effective oral health interventions to help manage frailty at a national level.

## Conclusions

Our study findings highlighted poor oral health and inadequate oral hygiene practices are altogether linked to experiencing frailty, as demonstrated in both frailty models. We also found more than half of the adults at least 50 years old were *frail* or in *pre-frail* stages, and the majority were on poor oral hygiene practices. Increasing awareness of the importance of oral health in maintaining robustness is needed. More studies using prospective data to assess the effectiveness of oral health interventions in preventing frailty are required.

## Supplementary Information

**Additional file 1: Supplementary Table 1**. Operational definition of frailty phenotype. **Supplementary Table 2**. Operational definition of 44-item frailty index. **Supplementary Table 3**. Comparison of included vs. excluded population (age ≥ 50 years). **Supplementary Table 4**. Sensitivity analyses for periodontal disease and frailty. **Supplementary Table 5**. Sensitivity analyses for number of natural teeth and frailty.

## Data Availability

Data used in this study is publicly available for research purposes in a de-identified fashion at the official website of KNHANES: https://knhanes.kdca.go.kr/knhanes/eng/index.do.

## Abbreviations

CI: Confidence intervals
CPI: Community periodontal index
FI: Frailty index
FP: Frailty phenotype
IRB: Institutional Review Board
KDCA: Korea Disease Control and Prevention Agency
KNHANES: Korea National Health and Nutrition Examination Survey
MSA: Metropolitan statistical areas
NHANES: National Health and Nutrition Examination Survey
OR: Odds ratio
PD: Periodontal disease
SE: Standard error
US: United States
WHO: World Health Organization

## References

[1] Fassbender K, Fainsinger RL, Carson M, Finegan BA (2009). Cost trajectories at the end of life: the Canadian experience. J Pain Symptom Manag.

[2] Fried LP, Tangen CM, Walston J, Newman AB, Hirsch C, Gottdiener J (2001). Frailty in older adults: evidence for a phenotype. J Gerontol A Biol Sci Med Sci.

[3] Hoogendijk EO, Afilalo J, Ensrud KE, Kowal P, Onder G, Fried LP (2019). Frailty: implications for clinical practice and public health. Lancet.

[4] World Health Organization (2016). WHO Clinical Consortium on Healthy Ageing.

[5] Blodgett J, Theou O, Kirkland S, Andreou P, Rockwood K (2015). Frailty in NHANES: comparing the frailty index and phenotype. Arch Gerontol Geriatr.

[6] Searle SD, Mitnitski A, Gahbauer EA, Gill TM, Rockwood K (2008). A standard procedure for creating a frailty index. BMC Geriatr.

[7] Mañas LR, Fielding RA, Sieber C, Vellas B (2015). Determinants of frailty and longevity: are they the same ones?. Frailty: pathophysiology, phenotype and patient care.

[8] Hakeem FF, Bernabe E, Sabbah W (2019). Association between oral health and frailty: a systematic review of longitudinal studies. Gerodontology.

[9] Ramsay SE, Papachristou E, Watt RG, Tsakos G, Lennon LT, Papacosta AO (2018). Influence of poor Oral health on physical frailty: a population-based cohort study of older British men. J Am Geriatr Soc.

[10] Bassim C, Mayhew AJ, Ma J, Kanters D, Verschoor CP, Griffith LE, Raina P (2020). Oral health, diet, and frailty at baseline of the Canadian longitudinal study on aging. J Am Geriatr Soc.

[11] Hakeem FF, Bernabe E, Sabbah W (2021). Association between Oral health and frailty among American older adults. J Am Med Dir Assoc.

[12] Hajishengallis G, Chavakis T (2021). Local and systemic mechanisms linking periodontal disease and inflammatory comorbidities. Nat Rev Immunol.

[13] Sanz M, Marco Del Castillo A, Jepsen S, Gonzalez-Juanatey JR, D'Aiuto F, Bouchard P (2020). Periodontitis and cardiovascular diseases: consensus report. J Clin Periodontol.

[14] van der Maarel-Wierink CD, Vanobbergen JN, Bronkhorst EM, Schols JM, de Baat C (2013). The importance of oral health in (frail) elderly people – a review. Eur Geriatr Med.

[15] Klinge B, Hultin M, Berglundh T (2005). Peri-implantitis. Dent Clin N Am.

[16] Nomura Y, Kakuta E, Okada A, Otsuka R, Shimada M, Tomizawa Y (2020). Effects of self-assessed chewing ability, tooth loss and serum albumin on mortality in 80-year-old individuals: a 20-year follow-up study. BMC Oral Health.

[17] Albani V, Nishio K, Ito T, Kotronia E, Moynihan P, Robinson L (2021). Associations of poor oral health with frailty and physical functioning in the oldest old: results from two studies in England and Japan. BMC Geriatr.

[18] KDCA. Korea National Health & Nutrition Examination Survey. https://knhanes.kdca.go.kr/knhanes/eng/index.do. Accessed 20 Jul 2021.

[19] Borges MK, Aprahamian I, Romanini CV, Oliveira FM, Mingardi SVB, Lima NA (2021). Depression as a determinant of frailty in late life. Aging Ment Health.

[20] Kim DH, Schneeweiss S, Glynn RJ, Lipsitz LA, Rockwood K, Avorn J (2018). Measuring frailty in Medicare data: development and validation of a claims-based frailty index. J Gerontol A Biol Sci Med Sci.

[21] Song X, Mitnitski A, Rockwood K (2010). Prevalence and 10-year outcomes of frailty in older adults in relation to deficit accumulation. J Am Geriatr Soc.

[22] Shin YU, Lim HW, Hong EH, Kang MH, Seong M, Nam E (2017). The association between periodontal disease and age-related macular degeneration in the Korea national health and nutrition examination survey: a cross-sectional observational study. Medicine (Baltimore).

[23] World Health Organization (1997). Oral health surveys: basic methods.

[24] Kim ES, Kim BI, Jung HI (2021). Age, period and cohort trends in oral health status in south Korean adults. Community Dent Oral Epidemiol.

[25] Swenor BK, Lee MJ, Tian J, Varadaraj V, Bandeen-Roche K (2020). Visual impairment and frailty: examining an understudied relationship. J Gerontol A Biol Sci Med Sci.

[26] Cesari M, Gambassi G, van Kan GA, Vellas B (2014). The frailty phenotype and the frailty index: different instruments for different purposes. Age Ageing.

[27] Kingman A, Albandar JM (2000). Methodological aspects of epidemiological studies of periodontal diseases. Periodontol.

[28] Beltrán-Aguilar ED, Eke PI, Thornton-Evans G, Petersen PE (2000). Recording and surveillance systems for periodontal diseases. Periodontol.

[29] Lee E, Lee SW (2019). Prevalence of periodontitis and its association with reduced pulmonary function: results from the Korean National Health and nutrition examination survey. Medicina (Kaunas).

[30] Won CW, Lee S, Kim J, Chon D, Kim S, Kim CO (2020). Korean frailty and aging cohort study (KFACS): cohort profile. BMJ Open.

[31] Fan J, Yu C, Guo Y, Bian Z, Sun Z, Yang L (2020). Frailty index and all-cause and cause-specific mortality in Chinese adults: a prospective cohort study. Lancet Public Health.

[32] Paw CA, MJ, de Jong N, Schouten EG, Hiddink GJ, Kok FJ. (2001). Physical exercise and/or enriched foods for functional improvement in frail, independently living elderly: a randomized controlled trial. Arch Phys Med Rehabil.

[33] Faber MJ, Bosscher RJ, Paw CA, MJ, van Wieringen PC. (2006). Effects of exercise programs on falls and mobility in frail and pre-frail older adults: a multicenter randomized controlled trial. Arch Phys Med Rehabil.

[34] Rockwood K, Song X, Mitnitski A (2011). Changes in relative fitness and frailty across the adult lifespan: evidence from the Canadian National Population Health Survey. CMAJ.

[35] KIHASA (2017). Community-based senior-friendly integrated medical service provision model.

