# Supplementary material for: Association between oral health and frailty: results from the Korea National Health and Nutrition Examination Survey
Source: BMC Geriatr. 2022 Apr 27;22:369. doi: 10.1186/s12877-022-02968-x (PMC9044774; doi:10.1186/s12877-022-02968-x)
Supplement: Supplementary file 1 — Additional file 1: Supplementary Table 1. Operational definition of frailty phenotype. Supplementary Table 2. Operational definition of 44-item frailty index. Supplementary Table 3. Comparison of included vs. excluded population (age ≥ 50 years). Supplementary Table 4. Sensitivity analyses for periodontal disease and frailty. Supplementary Table 5. Sensitivity analyses for number of natural teeth and frailty. [file 12877_2022_2968_MOESM1_ESM.pdf]

Association Between Oral Health and Frailty: Results from the Korean National Health and Nutrition Examination Survey

Hyunjoo Kim[a], Euni Lee[a,*], Seok-Woo Lee[b,*]

Running title: Oral health and frailty in Korean older adults

[a] College of Pharmacy & Research Institute of Pharmaceutical Sciences, Seoul National University, Seoul, Republic of Korea

[b] Department of Dental Education and Periodontology, School of Dentistry, Dental Science Research Institute, Chonnam National University, Gwangju, Republic of Korea

[*]**Corresponding author:**

Euni Lee, Pharm.D. Ph.D.

College of Pharmacy, Seoul National University, 1 Gwanak-ro, Gwanak-gu, Seoul, 08826, Republic of Korea (E-mail: eunilee@snu.ac.kr)

[*]**Co-corresponding author:**

Seok-Woo Lee, DDS, Ph.D.

Department of Dental Education and Periodontology, School of Dentistry, Dental Science Research Institute, Chonnam National University, Gwangju, 61186, Republic of Korea (E-mail: swlee@chonnam.ac.kr)

Keywords: frailty, oral health, health services for the aged, tooth loss, periodontal diseases

Supplementary Table 1. Operational definition of frailty phenotype

No.	Criteria	Definition	Collection method
1	**Unintentional weight loss**	Selected weight loss of 3 kg or more in the prior year, and selected 'Yes' for "I did not attempt to control my weight" or "I tried to gain weight".	Survey
2	**Weakness**	Measured grip strength, lowest 20% (stratified by gender, body mass index quartile).	Direct measurement
3	**Exhaustion**	Selected '7 days or more', or 'Almost every day' in PHQ-9 Depression Test item # 4 ("For the past two weeks, I had symptoms of exhaustion, loss of energy").	Survey
4	**Slowness**	Due to unavailability of gait speed data, this criterion was replaced by self-reported walking disability.<br><br>Selected 'Some problem walking about' or 'Confined to bed' in the EQ-5D-3L mobility domain.	Survey
5	**Low physical activity**	Metabolic equivalents (MET)-minutes/week, lowest 20% (stratified by sex). MET derived from the Global Physical Activity Questionnaire.	Survey

Supplementary Table 2. Operational definition of 44-item frailty index

No.	Criteria	Scoring
1	**Ever diagnosed with stroke**	0= No; 1=Yes
2	**Ever diagnosed with MI**	
3	**Ever diagnosed with angina pectoris**	
4	**Ever diagnosed with cancer (other than skin cancer)**	
5	**Current treatment of depression**	
6	**Ever diagnosed with rheumatic arthritis**	
7	**Ever diagnosed with osteoarthritis**	
8	**Ever diagnosed with thyroid illness**	
9	**Ever diagnosed with hepatitis B**	
10	**Current treatment of asthma**	
11	**Anemia***	0=No; 1=Yes (M: Hemoglobin [g/dL] <13, F (non-pregnant): <12; F (pregnant): <11)
12	**Low weight***	0=Not low; 1=Low (BMI <18.5 kg/m^2)
13	**Obese***	0=Not obese (<23); 0.5=Slightly obese (≥25 and <30); 0.25=Borderline obese (≥23 and <25); 0.75=Obese (≥30 and <35); 1=Most obese (BMI ≥35 kg/m^2)
14	**Irregular heart rate***	0=No; 1=Yes
15	**Creatinine clearance***	0=Not low; 1=Low (Cockcroft-Gault CrCl <60 mL/min)
16	**Coughed almost every day, continuously for at least 3 months, within 1 year**	0=No; 1=Yes
17	**Hematocrit***	0=Not low; 1=Low (≤24%)
18	**White blood cell count***	0=4~10 thous/uL (inclusive); else 1
19	**Red blood cell count***	0=M: 4.2~6.3 million cells/uL, F: 4.0~5.4 (inclusive); else 1
20	**Platelet**	0=150~450 thous/uL (inclusive); else 1
21	**High-sensitivity C-reactive protein**	0= <1 mg/L; 0.5= 1~3 (inclusive); 1= >3
22	**Poor hearing**	0=No; 1=Yes
23	**EQ5D: Difficulty walking**	0= No; 0.5=A little; 1=Severe
24	**EQ5D: Difficulty self-management**	
25	**EQ5D: Difficulty ADL**	
26	**EQ5D: Pain**	
27	**Poor self-reported health**	0=Very good-normal; 1=Bad-very bad
28	**Any current restrictions in daily living/social activity due to illness, physical or mental disorder**	0= No; 1=Yes
29	**Any stays in bed almost all-day last month**	0= No; 1=Yes
30	**Unintentional weight loss (more than 3 kg) within 1 year**	0= No; 1=Yes

**31**	**Weak grip strength (lowest 20% stratified by sex, body mass index quartile)**	0= No; 1=Yes
**32**	**Low physical activity (lowest 20%, stratified by sex)**	0= No; 1=Yes
**33**	**Little interest/pleasure in doing things**	(In the last two weeks) 0= Not at all; 0.5=Several days; 1=More than half the days or nearly every day
**34**	**Trouble falling or staying asleep, or sleeping too much**	
**35**	**Feeling tired or having little energy**	
**36**	**Poor appetite or overeating**	
**37**	**Trouble concentrating on things, such as reading the newspaper or watching television**	
**38**	**Hypertension***	0=No (SBP<120 and DBP<80); 0.5=Borderline (SBP≥120 or <140 or DBP≥80 or <90); 1=Yes (SBP≥140 mmHg or DBP≥90 or taking anti-hypertensive)
**39**	**Hypotension***	0=No; 1=Yes (SBP<90 mmHg or DBP<60)
**40**	**Hypercholesteremia***	0=No; 1= Yes (≥240 mg/dL after fasting 8 or more hours, or taking lipid control medications)
**41**	**High-density lipoprotein (HDL)***	0=No; 1=Low (<40 mg/dL after fasting 8 or more hours)
**42**	**Triglyceride***	0=No; 1=High (≥200 mg/dL after fasting 12 or more hours)
**43**	**Low-density lipoprotein (LDL)* (mg/dL)**	0=No; 1=High (≥160 mg/dL)
**44**	**Diabetes***	0=No (not 1 or 2, and <100); 0.5=Borderline (not 1, and ≥100 and <126); 1==Yes (Glucose ≥126 mg/dL after fasting 8 or more hours, or diagnosed with diabetes by a doctor or taking glucose-lowering medication or administering insulin)

* Clinical examination

Supplementary Table 3. Comparison of included vs. excluded population (age ≥ 50 years)

**Characteristics**		**Total**	**Frailty Phenotype** Included population	**Frailty Phenotype** Excluded population	***p***	**Frailty Index** Included population	**Frailty Index** Excluded population	***p***
Total		16295	4156 (22.37)	12139 (77.63)	-	15073 (91.16)	1222 (8.84)	-
Frailty phenotype	Robust	2059	2059 (53.79)	-	-	2059 (53.79)	-	-
	Pre-frail	1892	1892 (41.83)	-		1892 (41.83)	-	
	Frail	205	205 (4.38)	-		205 (4.38)	-	
Frailty index	Robust	4570	1473 (39.20)	3097 (31.54)	< 0.001	4570 (33.23)	-	-
	Pre-frail	8727	2397 (54.19)	6330 (56.26)		8727 (56.02)	-	
	Frail	1776	286 (6.61)	1490 (12.19)		1776 (10.74)	-	
Periodontal disease	No	7938	2196 (56.44)	5742 (53.91)	0.099	7479 (54.67)	459 (54.33)	0.880
	Yes	6482	1646 (43.56)	4836 (46.09)		6078 (45.33)	404 (45.67)	
Number of natural teeth, weighted mean (SE)		21.11	22.37 (0.18)	20.76 (0.12)	< 0.001	21.31 (0.10)	18.74 (0.39)	< 0.001
Age (years), weighted mean (SE)		63.06	61.68 (0.23)	63.27 (0.14)	< 0.001	62.94 (0.12)	64.23 (0.38)	0.001
Sex	Males	6972	1846 (46.96)	5126 (45.17)	0.086	6405 (44.69)	567 (50.35)	0.002
	Females	9323	2310 (53.04)	7013 (54.83)		8668 (55.31)	655 (49.65)	
Diet quality	Not poor	7523	1405 (53.33)	6118 (50.59)	< 0.001	6851 (56.03)	672 (60.71)	0.013
	Poor	8772	2751 (46.67)	6021 (27.04)		8222 (43.97)	550 (39.29)	
Living in rural areas	No	7461	2105 (50.72)	5356 (44.17)	0.032	6936 (45.87)	525 (44.49)	0.566
	Yes	8834	2051 (49.28)	6783 (55.83)		8137 (54.13)	697 (55.51)	
Low household income	No	6929	2028 (52.87)	4901 (44.49)	< 0.001	6524 (46.98)	405 (38.35)	< 0.001
	Yes	9261	2116 (47.13)	7145 (55.51)		8486 (53.02)	775 (61.65)	
Education (years)	≥ 9 years	8803	1345 (29.29)	4814 (41.53)	< 0.001	8802 (61.71)	1 (23.44)	0.090
	< 9 years	6159	2807 (70.71)	5996 (58.47)		6153 (38.29)	6 (76.56)	
Living alone	No	12107	3272 (81.05)	8835 (74.78)	< 0.001	11282 (76.51)	825 (70.89)	0.001
	Yes	4165	882 (18.95)	3283 (25.22)		3780 (23.49)	385 (29.11)	
Type of national health insurance	Self/workplace	15163	3997 (96.35)	11166 (92.62)	< 0.001	14119 (94.11)	1044 (87.50)	< 0.001
	Others	1132	159 (3.65)	973 (7.38)		954 (5.89)	178 (12.50)	
Smoking history	Never	9633	2613 (61.35)	7020 (59.42)	0.088	9292 (60.65)	341 (51.75)	0.001
	Past/current	5864	1538 (38.65)	4326 (40.58)		5583 (39.35)	281 (48.25)	
Frequent alcohol drinking	No	6181	1494 (34.25)	4687 (38.48)	< 0.001	5910 (37.51)	271 (39.30)	0.415
	Yes	9345	2660 (65.75)	6685 (61.52)		8987 (62.49)	358 (60.70)	
Number of comorbidities	< 2	8986	2315 (59.59)	6671 (59.20)	0.757	7892 (55.35)	1094 (92.78)	< 0.001
	≥ 2	7309	1841 (40.41)	5468 (40.80)		7181 (44.65)	128 (7.22)	
Brushed after breakfast, lunch, and dinner	No	12211	2871 (69.75)	9340 (76.82)	< 0.001	11145 (73.90)	1066 (86.94)	< 0.001
	Yes	4084	1285 (30.25)	2799 (23.17)		3928 (26.10)	156 (13.06)	
Currently using gargles	No	12199	3012 (73.07)	9187 (81.00)	< 0.001	11682 (78.51)	517 (80.30)	0.408
	Yes	3291	1139 (26.93)	2152 (19.00)		3184 (21.49)	107 (19.70)	

Unweighted frequencies (weighted percentage) shown, otherwise indicated. Missing data not shown. *p*-values from chi-square test or ANOVA.

Supplementary Table 4. Sensitivity analyses for periodontal disease and frailty

Characteristics	Sensitivity analysis 1 (FP) Age ≥ 50 years	Sensitivity analysis 2 (FP) Age ≥ 65 years	Sensitivity analysis 3 (FI) Age ≥ 65 years
Periodontal disease	0.993 (0.819-1.203)	0.932 (0.722-1.202)	0.973 (0.854-1.109)
Older	**1.045 (1.033-1.056)**	**1.128 (1.097-1.160)**	**1.147 (1.130-1.163)**
Female	**1.702 (1.405-2.061)**	**1.579 (1.220-2.043)**	**2.027 (1.778-2.310)**
Poor diet quality	0.998 (0.824-1.209)	**1.397 (1.083-1.802)**	0.970 (0.854-1.103)

Adjusted odds ratio (95% confidence interval) for frailty (outcome variable) shown. Bold values denote statistical significance.
Sensitivity analysis 1: Frailty phenotype criteria replaced, 'unintentional weight loss ≥ 3 kg' → 'current BMI ≤ 18.5 kg/m^2'
Sensitivity analysis 2: Frailty phenotype analysis population modified, adults aged ≥ 50 years → ≥ 65 years.
Sensitivity analysis 3: Frailty index analysis population modified, adults aged ≥ 50 years → ≥ 65 years.

Supplementary Table 5. Sensitivity analyses for number of natural teeth and frailty

**Characteristics**	**Sensitivity analysis 1 (FP)**	**Sensitivity analysis 2 (FP)**	**Sensitivity analysis 3 (FI)**
	**Age ≥ 50 years**	**Age ≥ 65 years**	**Age ≥ 65 years**
More number of natural teeth	**0.972 (0.962-0.983)**	**0.970 (0.957-0.983)**	**0.985 (0.978-0.992)**
Older	**1.037 (1.025-1.049)**	**1.117 (1.087-1.147)**	**1.141 (1.126-1.156)**
Female	**1.703 (1.415-2.051)**	**1.567 (1.232-1.993)**	**1.969 (1.739-2.229)**
Poor diet quality	1.011 (0.839-1.219)	**1.323 (1.034-1.692)**	0.965 (0.856-1.089)

Adjusted odds ratio (95% confidence interval) for frailty (outcome variable) shown. Bold values denote statistical significance.
Sensitivity analysis 1: Frailty phenotype criteria replaced, 'unintentional weight loss ≥ 3 kg' → 'current BMI ≤ 18.5 kg/m^2'
Sensitivity analysis 2: Frailty phenotype analysis population modified, adults aged ≥ 50 years → ≥ 65 years.
Sensitivity analysis 3: Frailty index analysis population modified, adults aged ≥ 50 years → ≥ 65 years.
